# An Integrated Psycho-Sociological Perspective on Public Employees’ Motivation and Performance

**DOI:** 10.3389/fpsyg.2019.00036

**Published:** 2019-01-22

**Authors:** Alina Ciobanu, Armenia Androniceanu, George Lazaroiu

**Affiliations:** ^1^Department of Administration and Public Management, Bucharest Academy of Economic Studies, Bucharest, Romania; ^2^Department of Social-Human Sciences, Spiru Haret University, Bucharest, Romania

**Keywords:** human resource management practices, performance, social exchange theory, public organizations, public service motivation

## Abstract

In the context of profound social, economic and financial changes, private and public organizations managers turn their attention towards the most valuable resource they have – the human resource (HR), the one that can ensure increased organizational performance. Using adequate HR policies and practices, organizations can build a positive work environment that sustains employees’ development, encourages communication, innovation, and pro-active attitudes and behaviors. However, managerial practices specific to the private sector do not seem to be the solution to the problems public institutions deal with. While specialists have mainly explored the link between human resources management (HRM) and performance in the private sector, due consideration ought to be paid to the particularities of this relationship in the public sector as well, and to the factors that influence public employees’ motivation and determine their level of performance. The purpose of our paper is to show that HRM in public institutions should be approached in a manner that focuses more on the work motivation theory, specifically on the psycho-sociological profile of the public employee, and argue that higher performance can be achieved by establishing a social exchange relationship between managers and the members of their teams. A supportive work environment positively impacts upon public employees’ performance, self-efficacy and job satisfaction, even though they are mainly driven in performing their tasks by intrinsic motivators and devotion to public values and interest.

## Introduction

In a period characterized by budgetary constraints, layoffs and frequent reorganizations, when increased emphasis is placed on efficiency and cost-effectiveness, public organizations need to put in place human resources (HR) practices that increase employees’ engagement and commitment in order to achieve enhanced levels of performance (Giauque et al., 2013; Albrecht et al., 2015). They have to go beyond recent evolutions, such as the New Public Management or the adoption of private sector managerial practices (Popescu Ljungholm, 2017), and adjust human resources management (HRM) policies to the attitudes and behaviors specific to public employees.

The relationship between HRM and performance has been the focus of specialists’ intensive research in the area for the last decades (Nishii et al., 2008; Saridakis et al., 2016), but in spite of all efforts it continues to be a complex and unclear link (Powell et al., 2014). It was often referred to as the “black box” due to the lack of clear understanding and empirical evidence explaining how the utilization of HR practices leads to organizational performance, as well as to the theoretical and methodological challenges raised by its study (Albrecht et al., 2015; Latorre et al., 2016). Recent research has examined the influence of HR practices upon organizational performance through several employee level mechanisms including job satisfaction, affective commitment, organizational citizenship behavior (OCB), psychological contract or perceived organizational support and social exchange (Alfes et al., 2013; Muduli, 2015; Latorre et al., 2016; Reimann et al., 2017).

Through our endeavor, we contribute to the academic discussion regarding individual mechanisms that influence public employees’ performance and assert that by putting in place the right association of HR practices, increased levels of commitment, dedication and absorption in work can be achieved. Considering the intrinsic nature of public employees’ motivation, public organizations can establish a social exchange relationship with their employees, show them the intention to invest in their well-being, and create a healthy work environment that benefits both parties.

The objective of our paper is to reveal the importance of investigating and considering the psychological profile of the public employee when decisions are made regarding the HR practices to be put in place in order to stimulate their commitment to the organization and to achieve enhanced performance. Unlike most specialists who believe that HR performance is primarily influenced by managers’ motivation systems, we consider that it is significantly shaped by managers’ ability to grasp and take into account the employees’ psychological profile (temperament, professional and individual skills, aspirations, concentration ability, stress resistance, etc.) when setting objectives or when evaluating the results obtained.

## Motivational Attitudes and Behaviors Specific to Public Employees

Public service motivation (PSM) represents a complex concept, one of the most studied and debated topics in the areas of public personnel management and public administration (Perry and Vandenabeele, 2015). The concept is one of an interdisciplinary nature given by the use of psychological principles in public administration, which has determined specialists to recently refer to it as “the precursor of behavioral public administration” (Vandenabeele et al., 2018).

Public service motivation represents the form of employee motivation specific to the public sector and is considered to be the most relevant concept when describing how public personnel’s motivation works (Giauque et al., 2013). PSM is defined as a pro-social value that encourages employees to engage in behaviors that are beneficial to the community or the society (Mostafa et al., 2015), combining intrinsic drivers such as altruism, compassion, commitment to public values, mission, and interest (Ritz et al., 2016). Considering that public employees perform to be useful to someone else, more willingly than to themselves, ponder on working to be of service to different persons, and not to themselves, and grasp their work as assisting other individuals, they undergo more work meaningfulness (Allan et al., 2018).

Public employees’ performance at the workplace is influenced by intrinsic motivational factors, such as responsibility, autonomy, interesting and important work, contribution, or fairness (Ciobanu and Androniceanu, 2015; Piatak, 2016). Maintaining high levels of engagement among public employees largely depends on the motivational potential of the work context, which derives from task variety and the meaningfulness of the tasks performed (Allan et al., 2018). Hierarchical ascendancy and the lack of organizational objective particularity may have adverse repercussions on the PSM–job satisfaction link (Kjeldsen and Hansen, 2018). Individuals with relevant levels of PSM have more lasting job satisfaction in contrast to their low-PSM fellows (Breaugh et al., 2018).

Employees connected to their tasks are highly engaged individuals, who allocate their full energy to performing their duties and display OCB’s that allow them to effectively complete their tasks. Engagement encompasses job attitudes such as job satisfaction, job involvement, and organizational commitment, and is a direct predictor of employee effectiveness (Mackay et al., 2017). It represents a work-related state of mind characterized by feelings of vigor, fulfillment, enthusiasm, dedication, and absorption in work (Eldor and Vigoda-Gadot, 2017; Shanafelt and Noseworthy, 2017). In performance terms, engaged employees put extra effort into their work to contribute to organizational success, aiming to align their competences to attain organizational goals. The effect of PSM on self-assessed service quality is longer-lasting for public personnel having more emotional intelligence (Levitats and Vigoda-Gadot, 2017), while its link to particular perceived performance is effective when the societal impact potential is significant (van Loon et al., 2018).

Human resource practices represent a reflection of the organizational environment surrounding public employees and play a particularly important role in shaping and nurturing PSM. Through the appropriate mix of HR practices public institutions can develop a supportive work environment, boost employees’ engagement and intrinsic motivation, and ensure that the organizational mission is fulfilled.

## Employees’ Perception of the HR Practices is Influenced by Personal Traits

In the recent decades, work relations have registered a constant evolution from collectivism to an individual-based approach especially encouraged by performance management, which pays particular attention to personal traits in the seek for increased HR performance. In addition to individual variables such as education, professional training and experience, personality traits also influence individual experience of job resources, safety, engagement, and employee outcomes (job satisfaction, commitment, absence and turnover intention) and determine the employee to behave in different ways (Alfes et al., 2013; Albrecht et al., 2015; Pera, 2017). Employees undergo unstable levels of commitment when carrying out their work, and may shape their own degrees of commitment. Job crafting constitutes a valuable bottom-up approach to enhance work engagement, as it raises the meaning of work and the adjustment between employee and organization (Bakker and Albrecht, 2018).

The relationship between HR practices and individual outcomes is strongly influenced by employees’ perception. Based on the psychological theories concerning individual differences in cognition, affect, and motivation, Nishii et al. (2008) showed that employees do not respond uniformly to the same set of HR practices. The implementation of high performance HR practices may not always be perceived in a positive light as employees can receive it as a signal that increased effort is expected on their behalf, and that increased performance is pursued rather than their well-being (Jensen et al., 2013). Moreover, if employees feel that the effort they put in is not reciprocated by the organization, they may experience feelings of strain. On the other hand, high commitment HR practices positively influence employees’ well-being and signal that they are seen as valuable resources.

However, the stringent constraint to obtain increased performance with reduced costs generates pressure for public institutions, which tend to pay less attention to the well-being of their workforce. In this context, Guest (2017) proposes to approach HRM in a manner that would create mutual benefits for organizations and employees. Using the model of public goods game, Chen et al. (2016) argue that individual and collective benefits are the highest provided that all interested parties adopt the cooperative strategy. Therefore, by implementing practices that enhance employees’ well-being and create a positive employment relationship, based on trust and cooperation, both individual and organizational performance will be improved, thus generating mutual gains. Di Fabio (2017) considers that a further step should be taken in employees’ motivation and proposes to shift from the motivational paradigm, which considers intrinsic, extrinsic or lack of motivation, to the paradigm based of meaningfulness, which represents the intrinsic motivational energy – the key to the development, performance and health for both the employee and the organization. Considering the specifics of public employees’ motivation, a HRM approach based on meaningfulness can represent the change in the way HR are managed in public institutions as well as the change in people’s attitudes (Androniceanu and Popescu, 2017) that will contribute to creating a stimulating work environment. Both academicians and practitioners are turning to spirituality and karma to solve modern day HR challenges and report these concepts as necessary determinants of employee commitment, job satisfaction and work-life balance satisfaction (Garg, 2017).

## Using HR Practices to Enhance Public Employees’ Motivation

There are though specialists who consider that too much emphasis is placed on personal values, while the organizational context is disregarded (Andrews, 2016) and have turned to the analysis of the influence high-performance HR practices produce upon public employees’ motivation (Gould-Williams, 2016). Traditionally high-performance HR practices have been defined as a group of coherent, interrelated, mutually reinforcing HRM policies and practices, designed to enhance individual and organizational performance by enhancing employees’ competencies, motivation, commitment, and productivity (Muduli, 2015). More recent definitions have gone beyond the classical approach of “high-performance HR practices,” which focuses on the developmental component of training and personal development, job design or compensation, and have given the concept a psychological dimension. Employees are encouraged to invest additional, discretionary, time and effort at their workplace through motivation-enhancing and opportunity to participate practices, such as incentive payment, performance-related reward, flexible work schedules, participation programs, discretion, and authority on the job (Saridakis et al., 2016). This type of HR practices stimulates employees to exchange job-related information, enhances their creativity, develops their abilities to innovate and generate new ideas (Fu et al., 2015).

High-performance HR practices have been referred to either as high-performance work systems (Fu et al., 2015; Van De Voorde and Beijer, 2015), high commitment HR practices (Giauque et al., 2013; Mostafa et al., 2015) or high-involvement HR practices (Batt and Colvin, 2011). Gould-Williams (2016) criticizes the use of these concepts in an interchangeable manner and argues that each of them is appropriate to a certain organizational context, while their implementation is likely to be driven by different motives. Through high-performance HR practices investments are made in employees, who are considered valuable strategic resources in the pursuit of superior performance achievement. This approach has been mainly adopted by private companies, whose functioning is guided by the goal of obtaining competitive advantage. Through high commitment HR practices, besides improved performance, employees’ commitment towards the organization is also sought. Considering the principles of the PSM theory, high commitment HR practices ought to generate positive outcomes upon public employees’ behaviors since their individual objectives overlap the organizational ones, that is to serve the public interest. High involvement HR practices imply training employees to get involved in the decision making process, putting in place methods that allow them to carry out tasks without close supervision and encourage self-determined behaviors (Gould-Williams, 2016). The bureaucratic and hierarchical structure of public institutions may hamper the development of such behaviors in the employees. However, further research is necessary before drawing a conclusion regarding the appropriateness of implementing this type of HR practices in the public sector.

**FIGURE 1:**
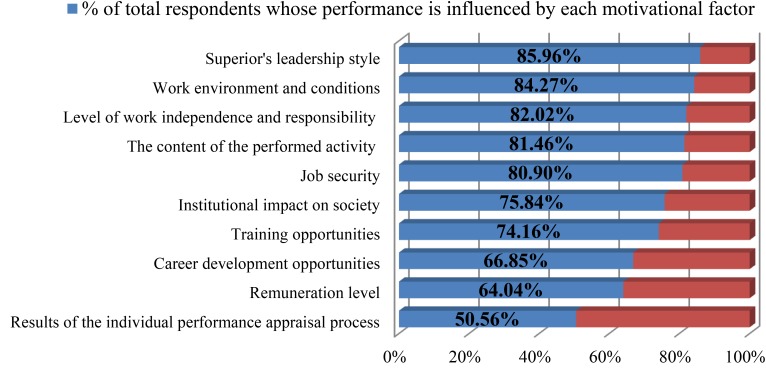
Motivational factors that influence civil servants’ performance in the workplace. Source: Adapted after Ciobanu and Androniceanu (2018).

Through the implementation of high commitment HR practices, organizations promote positive relations with their employees and show their intention to form a long-term social relationship (Latorre et al., 2016). This type of HR practices allows managers to show their concern for employees’ well-being, appreciation and recognition for their effort triggering positive attitudes and behaviors such as engagement, affective commitment, job satisfaction and OCB, which have been linked with enhanced organizational performance (Nica et al., 2017). Managers play an important part in shaping individuals’ work behavior and their contribution to achieving organizational goals, a conclusion supported by the results of the study of Ciobanu and Androniceanu (2018), which ranked *superior’s leadership style* as the motivational factor that exerts the highest influence upon civil servants’ work performance out of the 10 evaluated factors (Figure 1).

The numerical simulations performed by Chen et al. (2016) and Xia et al. (2017) indicate that individual reputation influences the level of cooperation in a social environment and show that by introducing the reputation effect in the traditional public goods game, the more influential cooperators will attract and determine more individuals to adopt their strategy. Furthermore, preponderant individuals with high reputation values ensure not only higher cooperation levels, but also prevent individuals from adopting the defector attitude, that is to act as free-riders or to refrain from making contributions to the group activities. In a public institution, managers are responsible to disseminate the mission and objectives of the organization among employees, to promote a cooperative work environment, to determine HR to use their competences and invest additional efforts into reaching departmental and organizational goals. Transposing the principles of evolutionary game theory (Chen et al., 2016; Xia et al., 2017) at the level of public institutions, we appreciate that managers with high reputation values will be able to establish long-term relationships between employees and organization using high-commitment HR practices, to determine them to adhere to the organizational mission and to get fully involved into meeting organizational goals.

## Drawing on the Social Exchange Theory to Achieve the Desired Attitudes and Behaviors in Public Employees

An increasing number of studies has turned to the social exchange theory to show that employees’ commitment and engagement exert significant influence upon their performance (Van De Voorde and Beijer, 2015; Zhong et al., 2016). The social exchange theory lies at the heart of the psychological contract and consists in a bidirectional relationship based on promises and obligations (Latorre et al., 2016). When the HR practices indicate employees the intention of the organization to invest in them, to support and provide them benefits and resources, and to enhance their well-being, they are likely to respond with positive attitudes and behaviors, develop an affective bond with the organization and feel motivated to work towards organizational goals (Mostafa et al., 2015; Khoreva and Wechtler, 2018).

The social exchange theory focuses more specifically on the employment relationship and the exchanges implied in that relationship (Latorre et al., 2016). At the center of the link between HRM and performance there is a positive employment relationship, from an employee perspective, which consists of a set of three perceptions closely connected with social exchange, namely the perceived organizational support, the psychological contract and job security (Latorre et al., 2016). The role of HR practices in the employment relationship is to signal and help communicate the content of the psychological contract, i.e., the promises for the future and contributions expected. High commitment HR practices have been associated with the perceptions generated by the social exchange relationship, namely perceived organizational support and a sense of job security, increased job satisfaction and commitment towards the organization (Kurtessis et al., 2017; Khoreva and Wechtler, 2018). A karmic approach of HRM offers managers the possibility to be more caring about the needs and fates of employees (Kollen, 2016).

Motivation emerges from a reliable long-term social exchange relationship (Giauque et al., 2013), based on fairness, a value which is more easily cultivated in a centralized work system, characterized by uniform rules, such as the government and public institutions, rather than in a decentralized HRM system (Prysmakova, 2016). Based on the principles that ground the social exchange theory, Mostafa et al. (2015) demonstrate that public employees’ perception of the high-performance HR practices influences PSM, which in turn is associated with two desired employee outcomes: affective commitment and OCB. Thus, when organizations signal their desire to invest in employees’ well-being and put into practice high-performance HR practices, employees reciprocate by showing positive work-related attitudes and behaviors, go beyond task performance, and carry out activities that are not contractually specified, benefiting the organization. PSM, organizational affinity, subjective organizational citizenship behavior norms, task interconnection, and procedural justice constitute significant precursors of government employees’ OCB (Shim and Faerman, 2017). The relationship between high-performance HR practices and employee attitudes and behaviors is based on an emotional mechanism and the use of this type of practices leads to increased job satisfaction and OCB in this particular context (Friedman and Gerstein, 2017; Mostafa, 2017).

The implementation of high-commitment HR practices in the public sector, specifically of the intrinsic HR practices, will consolidate employees’ engagement towards civic duties and their commitment to the organization and will stimulate them to invest extra effort and resources into their work. In this manner, public employees are signaled first of all that the organization is engaged in a long-term social exchange relationship and secondly are guaranteed job security, i.e., two important prerequisites of increased motivation, a psychological state nurtured by a reliable long-lasting relationship. This approach turns managers’ attention towards the *human* side of the HRM by placing the employee in the spotlight and outlining their individual psycho-sociological profile. Employees are no longer perceived as the strategic resources that contribute to achieving organizational objectives, but as persons who display organizational commitment behaviors when managers prove concern for theirwell-being.

## Conclusion

Our findings bring new insights on the relationship between HRM and performance in the public sector, suggesting that public employees’ commitment to the organization, creativity and efficiency in solving work-related matters can increase in a positive organizational support framework where the implemented HR practices are aimed at their well-being. The main gap identified by the review concerns the individual traits that come up in the relationship between HRM and performance in the case of public employees. Based on the psychological profile, public managers can design and put in place the association of HR practices that will generate a stimulating working climate and will help them achieve the much sought increased efficiency and performance in public organizations. The inferences of the advancements summarized in the previous sections of this article indicate that, by drawing on the principles of the social exchange theory, enhanced professional results can be achieved by individuals who find intrinsic motivation in the activity they perform. Intrinsic HR practices contribute to creating a supportive work environment in public institutions developing a positive relationship between employees and their organization, generating engagement, affective commitment, and job satisfaction. By designing and implementing HRM solutions and methods customized according to their employees’ needs and desires, public institutions will be able to ensure civil servants’ continuous development, will encourage initiative and pro-active attitude, and will create the premises to achieve organizational objectives (Compton and Meier, 2016).

The influence of the organizational environment, specifically of the high commitment HR practices upon public employees’ motivation, job satisfaction, commitment and level of performance, remains little explored by the literature in the area. Future research should focus on how to enhance the performance of public service personnel in connection with developments in big data analytics capabilities and artificial intelligence algorithms, and taking into account personal data privacy and consumer digital rights. Further developments on the subject should be grounded on the results of an empirical research aiming to identify the HR practices that produce the desired impact upon public employees’ attitudes and behaviors, that encourage their dedication towards the public interest and values, thus contributing to achieving organizational goals.

## Author Contributions

All authors listed have made a substantial, direct and intellectual contribution to the work, and approved it for publication.

## Conflict of Interest Statement

The authors declare that the research was conducted in the absence of any commercial or financial relationships that could be construed as a potential conflict of interest.
